# Head Motion in Diffusion Magnetic Resonance Imaging: Quantification, Mitigation, and Structural Associations in Large, Cross‐Sectional Datasets Across the Lifespan

**DOI:** 10.1002/hbm.70143

**Published:** 2025-02-11

**Authors:** Kurt G. Schilling, Karthik Ramadass, Viljami Sairanen, Michael E. Kim, Francois Rheault, Nancy Newlin, Tin Nguyen, Laura Barquero, Micah D'archangel, Chenyu Gao, Ema Topolnjak, Nazirah Mohd Khairi, Derek Archer, Lori L. Beason‐Held, Susan M. Resnick, Timothy Hohman, Laurie Cutting, Julie Schneider, Lisa L. Barnes, David A. Bennett, Konstantinos Arfanakis, Sophia Vinci‐Booher, Marilyn Albert, The BIOCARD Study Team, The Alzheimer's Disease Neuroimaging Initiative (ADNI), Aging Brain: Vasculature, Ischemia, and Behavior (ABVIB), Daniel Moyer, Bennett A. Landman

**Affiliations:** ^1^ Department of Radiology & Radiological Sciences Vanderbilt University Medical Center Nashville Tennessee USA; ^2^ Vanderbilt University Institute of Imaging Science Nashville Tennessee USA; ^3^ Department of Computer Science Vanderbilt University Nashville Tennessee USA; ^4^ Baby Brain Activity Center, Children's Hospital, Helsinki University Hospital and University of Helsinki Helsinki Finland; ^5^ Department of Radiology Kanta‐Häme Central Hospital Hämeenlinna Finland; ^6^ Medical Imaging and Neuroinformatic (MINi) Lab Universite de Sherbrooke Quebec Canada; ^7^ Department of Special Education Peabody College of Education and Human Development, Vanderbilt University Nashville Tennessee USA; ^8^ Vanderbilt Kennedy Center Nashville Tennessee USA; ^9^ Department of Electrical and Computer Engineering Vanderbilt University Nashville Tennessee USA; ^10^ Vanderbilt Memory & Alzheimer's Center, Vanderbilt University Medical Center Nashville Tennessee USA; ^11^ Vanderbilt Genetics Institute, Vanderbilt University Medical Center Nashville Tennessee USA; ^12^ Laboratory of Behavioral Neuroscience National Institute on Aging, National Institutes of Health Baltimore Maryland USA; ^13^ Department of Neurology Vanderbilt University Nashville Tennessee USA; ^14^ Rush Alzheimer's Disease Center, Rush University Medical Center Chicago Illinois USA; ^15^ Department of Biomedical Engineering Illinois Institute of Technology Chicago Illinois USA; ^16^ Department of Diagnostic Radiology Rush University Medical Center Chicago Illinois USA; ^17^ Department of Psychology and Human Development Peabody College, Vanderbilt University Nashville Tennessee USA; ^18^ Department of Neurology Johns Hopkins University School of Medicine Baltimore Maryland USA

## Abstract

Head motion during diffusion magnetic resonance imaging (MRI) scans can cause numerous artifacts and biases subsequent quantification. However, a thorough characterization of motion across multiple scans, cohorts, and consortiums has not been performed. To address this, we designed a study with three aims. First, we aimed to characterize subject motion across several large cohorts, utilizing 13 cohorts comprised of 16,995 imaging sessions (age 0.1–100 years, mean age = 63 years; 7220 females; 3175 cognitively impaired adults; 471 developmentally delayed children) to describe the magnitude and directions of subject movement. Second, we aimed to investigate whether state‐of‐the‐art diffusion preprocessing pipelines mitigate biases in quantitative measures of microstructure and connectivity by taking advantage of datasets with scan‐rescan acquisitions and ask whether there are detectable differences between the same subjects when scans and rescans have differing levels of motion. Third, we aimed to investigate whether there are structural connectivity differences between movers and non‐movers. We found that (1) subjects typically move 1–2 mm/min with most motion as translation in the anterior–posterior direction and rotation around the right–left axis; (2) Modern preprocessing pipelines can effectively mitigate motion to the point where biases are not detectable with current analysis techniques; and (3) There are no apparent differences in microstructure or macrostructural connections in participants who exhibit high motion versus those that exhibit low motion. Overall, characterizing motion magnitude and directions, as well as motion correlates, informs and improves motion mitigation strategies and image processing pipelines.

## Graphical abstract

We characterized motion during diffusion MRI scans across 16,995 image sessions and find that (1) modern preprocessing pipelines effectively mitigate motion to the point where biases are not detectable, and (2) that there are no apparent differences in microstructure or connectivity in volunteers who exhibit high motion versus those that exhibit low motion.

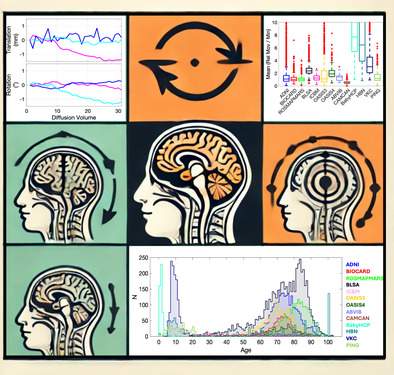

## 1 Introduction

Subject motion induces artifacts in MR images that have a downstream impact on image‐derived metrics. In structural studies (T1/T2‐weighted), motion results in misestimates of structure and geometry (Blumenthal et al. 2002; Savalia et al. 2017). In functional studies, motion causes image artifacts that confound analysis (Friston et al. 1996) and introduces spurious correlations in network analyses (Power et al. 2012). In diffusion studies, motion introduces misalignment between volumes, signal dropout, and erroneous signal attenuation (Anderson and Gore 1994) which can also bias measures and can artifactually create group differences (Yendiki et al. 2014). While different magnitudes, directions, and frequencies of motion can cause different artifacts, a thorough characterization of motion in terms of translation and rotation across a population has not been performed, particularly with diffusion magnetic resonance imaging (MRI) data. Thus, the first aim of this study is to characterize subject motion across several large cohorts. We utilize 13 cohorts comprised of 16,955 imaging sessions, ranging in age from 0.1 to 100 years, and quantify motion by describing magnitude and directions of subject movement, as well as the relationship between motion and age and cognitive status.

Although challenges with head motion in diffusion MRI have been recognized since its inception, most studies rely on registration‐based correction methods that (alone) do not fully mitigate the effects of head motion on diffusion‐derived measures (Yendiki et al. 2014). However, recent advances in image preprocessing incorporate not only global head motion correction, but also correct warping due to eddy currents, detect and replace outliers (Sairanen and Andersson 2023) for slice dropouts (Andersson et al. 2016), and incorporate susceptibility distortion correction. For example, the combination of TOPUP and EDDY tools from the fMRIB software library (FSL) are state‐of‐the‐art in diffusion preprocessing and have been broadly adopted by large imaging consortiums (Glasser et al. 2013; Alfaro‐Almagro et al. 2018). Recent studies suggest that these motion correction methods accurately detect and mitigate head motion in simulated data (Cieslak et al. 2024); however, it is unknown whether these advancements in image preprocessing and modeling remove biases introduced in empirically acquired data with motion artifacts. Thus, the second aim of this study is to investigate whether state‐of‐the art diffusion preprocessing and modeling pipelines mitigate biases in quantitative measures of microstructure and connectivity described by prior literature (Yendiki et al. 2014). To do this, we take advantage of two datasets that contain within‐session scan‐rescan acquisitions, selecting subjects that display the greatest differences in head motion between scan and rescan and analyzing these as if they were two separate cohorts—movers and non‐movers. Because these are the same subjects, any quantitative differences between these cohorts are artifacts introduced by motion.

Finally, several studies have found strong relationships between head motion and functional properties of the brain. For example, associations between head motion and executive function (Hausman et al. 2022), cognitive status (Geerligs et al. 2017), and inhibitory control and working memory (Bolton et al. 2020) have all been reported. Taking advantage of scan‐rescan data of the same subjects, Zeng et al. (Zeng et al. 2014) found large‐scale functional connectivity differences between individuals with high head motion (movers) and those with low motion (non‐movers) that were not found in intrasubject data, suggesting that motion‐associated differences in brain function are not just motion artifacts, but reflect true differences in functional organization. However, similar analyses have not yet been performed to explore possible microstructural and structural connectivity differences between movers and non‐movers. Thus, the third, and final, aim of this study is to investigate whether there are structural connectivity differences between movers and non‐movers. By classifying subjects as high and low‐motion individuals, we ask whether there are differences in microstructure or connectivity between these high and low‐motion cohorts.

## 2 Methods

### 2.1 Characterizing Motion During Diffusion MRI Scans

We used 13 large consortium datasets to study subject motion across a population aged 0–100 years. Specifically, we chose diffusion MRI data which involved collection of multiple volumes over several minutes and allowed characterizing motion: ADNI (*N* = 2521) (Jack Jr et al. 2008), BIOCARD (*N* = 974) (Albert et al. 2014), ROSMAP (*N* = 1973) (Bennett et al. 2018), BLSA (*N* = 5424) (Williams et al. 2019), ICBM (*N* = 140) (Crawford, Neu, and Toga 2016), OASIS3 (*N* = 1794) (Pamela et al. 2019), OASIS4 (*N* = 603) (Marcus et al. 2007), ABVIB (*N* = 63), CAMCAN (*N* = 305) (Taylor et al. 2017), BabyHCP (*N* = 494) (Howell et al. 2019), HBN (*N* = 1006) (Alexander et al. 2017), PING (Jernigan et al. 2016) (*N* = 431), and data collected by the Vanderbilt Kennedy Center (VKC) (*N* = 149). A visual summary of datasets, sample size, and age range is given as Figure S1.

Of note, two datasets, BLSA and BIOCARD, included scan‐rescan within session data, run consecutively within the same session. Datasets ADNI, BIOCARD, ROSMAPMARS, BLSA, OASIS3, and OASIS4 included cognitively normal (CN), cognitively impaired (CI), and subjects with Alzheimer's Disease (AD). Finally, the VKC dataset included children who all scored within the typically developing range on IQ tests; however, some were at risk for or had learning disabilities (LD) and/or other developmental disorders (e.g., ADHD). Boxplots of sample size and cohort size for these datasets is given in Figure S2.

Among the datasets were differing scan parameters (TR, TE, number of volumes, resolution) and varying diffusion gradient‐schemes, and hence different scan durations and likely different sensitivity to motion. Diffusion preprocessing was performed using the PreQual (Cai et al. 2021) end‐to‐end preprocessing pipeline to attenuate susceptibility, motion, and eddy currents. Importantly, this includes FSL's (Jenkinson et al. 2012) TOPUP and EDDY commands (Andersson, Skare, and Ashburner 2003). Specifically, EDDY was run with outlier detection and replacement (Andersson et al. 2016) (−repol flag), but all other parameters set as default.

**FIGURE 1:**
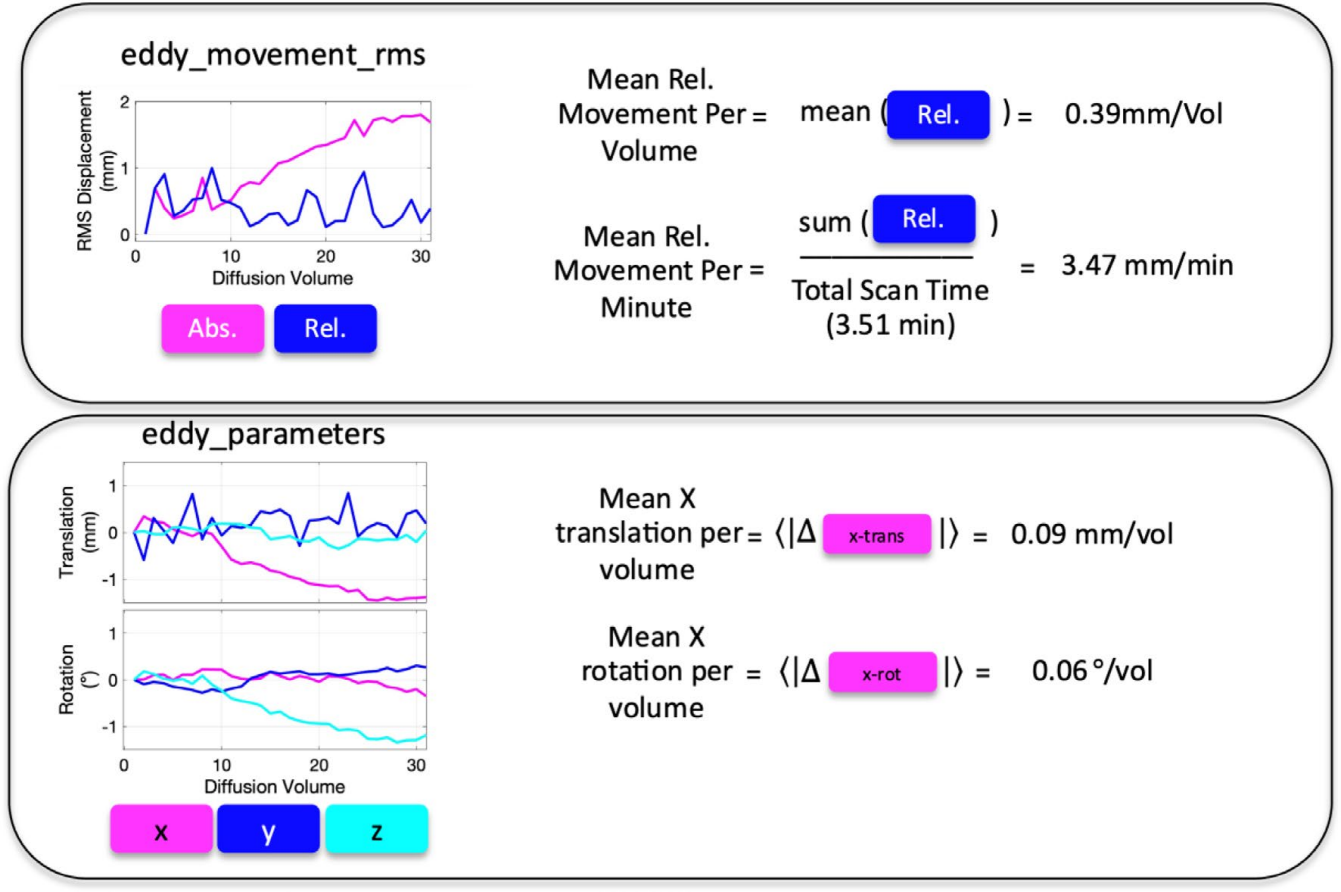
Derivation of head motion descriptors. All motion parameters were derived from output of FSL software's *eddy* algorithm: Eddy_movement_rms (top) and eddy_parameters (bottom) files. From eddy_movement_rms, the “mean relative movement per imaging volume” (units of mm/volume) was calculated as the mean of the relative RMS displacement. This measure can be normalized by total scan time to the “mean relative movement per minute” (units of mm/min). From eddy_parameters, measures of absolute translation and rotation are given with respect to the first imaging volume. To calculate the “mean translation per volume”, we take the mean of the absolute values of differences from volume to volume (units of mm/vol). This is repeated for translation along *x*, *y*, and *z* axes, as well as rotation around L/R, P/A, and I/S axis (units of degrees/vol).

From the preprocessing, two files of interest were output from which all motion descriptors were derived (Figure 1). The first is *eddy_movement_rms* in which as described in the FSL documentation (https://fsl.fmrib.ox.ac.uk/fsl/fslwiki/eddy/UsersGuide) is “a summary of the ‘total movement’ in each volume is created by calculating the displacement of each voxel and then averaging the squares of those displacements across all intracerebral voxels. The file has two columns where the first contains the RMS movement relative to the first volume and the second column the RMS relative to the previous volume.” Second is the eddy_parameters file, which is described as “…a text file with one row for each volume in [the image] and one column for each parameter. The first six columns correspond to subject movement starting with three translations followed by three rotations…”. From these two files, derived measures included relative mean displacement (mm), translation in Left/Right, Posterior/Anterior, Interior/Superior directions (mm), and rotation around L/R, P/A, I/S axis (radians). All measures were derived per imaging volume, and were also normalized to scan duration (i.e., mean displacement per minute) (See Figure 1 for plots and exemplar derivations).

This combined dataset was used to address the first aim. Specifically, these data were used to answer (1) How much do participants move during diffusion scans? (2) What kind of head motion, and in which direction, is most common during diffusion MRI scans? (3) Does motion get worse as the acquisition proceeds? (4) Is motion related to age cross‐sectionally? and (5) Is motion related to cognitive impairment cross‐sectionally?

Questions 1–4 were answered by simply displaying derived motion estimates (total displacement, 3 translations, 3 rotations, etc) for each dataset, and as a function of acquisition time or subject age. Question 5 was answered by comparing the CN, CI, and AD for datasets with these populations (ADNI, BIOCARD, ROSMAPMARS, BLSA, OASIS3, OASIS4) or comparing CN and LD for the VKC dataset, using the Wilcoxin rank sum test, a nonparametric test for two populations when samples are independent. *p*‐values were controlled for false discovery rates for multiple hypothesis testing using the method of Benjamini & Hochberg (Benjamini and Hochberg 1995).

### 2.2 Motion‐Induced Biases in Microstructure and Connectivity

BLSA and BIOCARD datasets were used to investigate motion‐induced biases in microstructure and structural connectivity due to the availability of scan‐rescan intra‐session scans. The mean relative movement per image volume was calculated for each scan and rescan, and these were classified, for each subject, into the “less‐motion” (“< motion”) and “more‐motion” (“> motion”) scan. The 120 individuals with the greatest difference in these two scans were selected for analysis. The less‐ and more‐motion scans were treated as two cohorts and analyzed. Any differences between these two cohorts are purely artifactual, as they are the same subjects within the same session, on a time scale much shorter than true biological changes in structural connectivity would be expected to occur.

For every scan, sets of white matter pathways were virtually dissected using the TractSeg (Wasserthal, Neher, and Maier‐Hein 2018) automated tractography algorithm. Briefly, TractSeg was based on convolutional neural networks and performed bundle specific tractography based on a field of estimated fiber orientations (Wasserthal, Neher, and Maier‐Hein 2018). We implemented the dockerized version at (https://github.com/MIC‐DKFZ/TractSeg), which generated fiber orientations using constrained spherical deconvolution with the MRtrix3 software (Tournier et al. 2019). Inputs were the preprocessed diffusion data (run with the–raw_diffusion_input flag), and TractSeg was run with default parameters and settings. TractSeg resulted in 71 bundles, including association, limbic, commissural, thalamic, striatal, and projection and cerebellar pathways.

For each white matter pathway, two sets of features were extracted. First, *microstructural* features were extracted by performing diffusion tensor analysis (Pierpaoli et al. 1996; Basser, Mattiello, and LeBihan 1994) (after extracting only *b*‐values *b* < 1500 s/mm^2^, if applicable), and extracting the averaged fractional anisotropy (FA), mean diffusivity (MD), axial diffusivity (AD), and radial diffusivity (RD) across each pathway. Second, *macrostructural* features of each pathway were extracted, including volume, volume of the endpoints, average and standard deviation of length, min/max length, and mean curvature.

Finally, *graph theory* analysis was applied to describe the connectome (Hagmann et al. 2008). This was performed by performing whole‐brain tractography, and assigning connectome edge weights based on nodes defined by the Desikan Killiany atlas (Desikan et al. 2006) (84 total nodes). Briefly, a group averaged response function was derived by averaging (25 randomly selected subjects) response functions (using *dwi2response dhollander* algorithm (Dhollander and Connelly 2016; Dhollander, Raffelt, and Connelly 2016)), fiber orientation distributions were reconstructed (using *dwi2fod msmt_csd* algorithm (Jeurissen et al. 2014)), tractography was performed (using *tckgen iFOD2* algorithm (Tournier, Calamante, and Connelly 2010)) using anatomical constraints and seeding from the white‐gray‐matter boundary until 10 million streamlines were generated (Smith et al. 2012). Finally, streamline weighting factors were determined (using *tcksift SIFT2* algorithm (Smith et al. 2015)) such that streamlines are modulated by a fiber density (i.e., the cross‐sectional area) as determined by the spherical deconvolution model. We note that all results presented generalize to both modulated (SIFT2 weights) and unmodulated (simply the number of streamlines between nodes) connectomes, thus, results are shown for the connectomes weighted by SIFT2 weights. The Brain Connectivity Toolbox (Rubinov et al. 2009) (From Mathworks, MatLab, v1.1.1.0) was used to calculate connectome edge density, path length, clustering coefficient, assortativity, and global network efficiency.

For microstructure, macrostructure, and connectomic measures, differences between low‐ and high‐motion datasets were assessed using the Wilcoxon signed rank test, a nonparametric test for two populations for paired observations. Due to multiple comparisons, all statistical tests were controlled by the false discovery rate at 0.05 to determine significance (Benjamini and Hochberg 1995).

These data were used to address the second aim. Specifically, we ask whether motion introduces biases in microstructure, macrostructure, or connectomic measures when processed with modern pipelines.

### 2.3 Structural Connectivity Differences Between Movers and Non‐Movers

BLSA and BIOCARD datasets were again used to investigate whether there are intersubject differences between movers and non‐movers. First, the mean relative movement per image volume was calculated for all scan sessions. From the distribution of movements, non‐movers were defined as subjects who (for all cross‐sectional and/or longitudinal sessions) had motion in the bottom 25th percentile. Similarly, movers were defined as those that consistently had head motion in the top 25th percentile.

Again, TractSeg was used to automatically segment the same association, projection, and commissural pathways. Microstructural features and macrostructural features were derived for each segmented bundle, and global connectome measures were similarly derived.

Differences between movers and non‐movers were assessed using a general linear model with fixed effects of group, age, and sex, where the effect of group (mover versus non‐mover) was the coefficient of interest (note covarying for age/sex was not used in the intrasubject analysis as it was the same subjects within each cohort). Again, due to multiple comparisons, all statistical tests were controlled by the false discovery rate at 0.05 to determine significance (Benjamini and Hochberg 1995).

## 3 Results

### 3.1 Characterizing Motion During Diffusion MRI Scans

**FIGURE 2:**
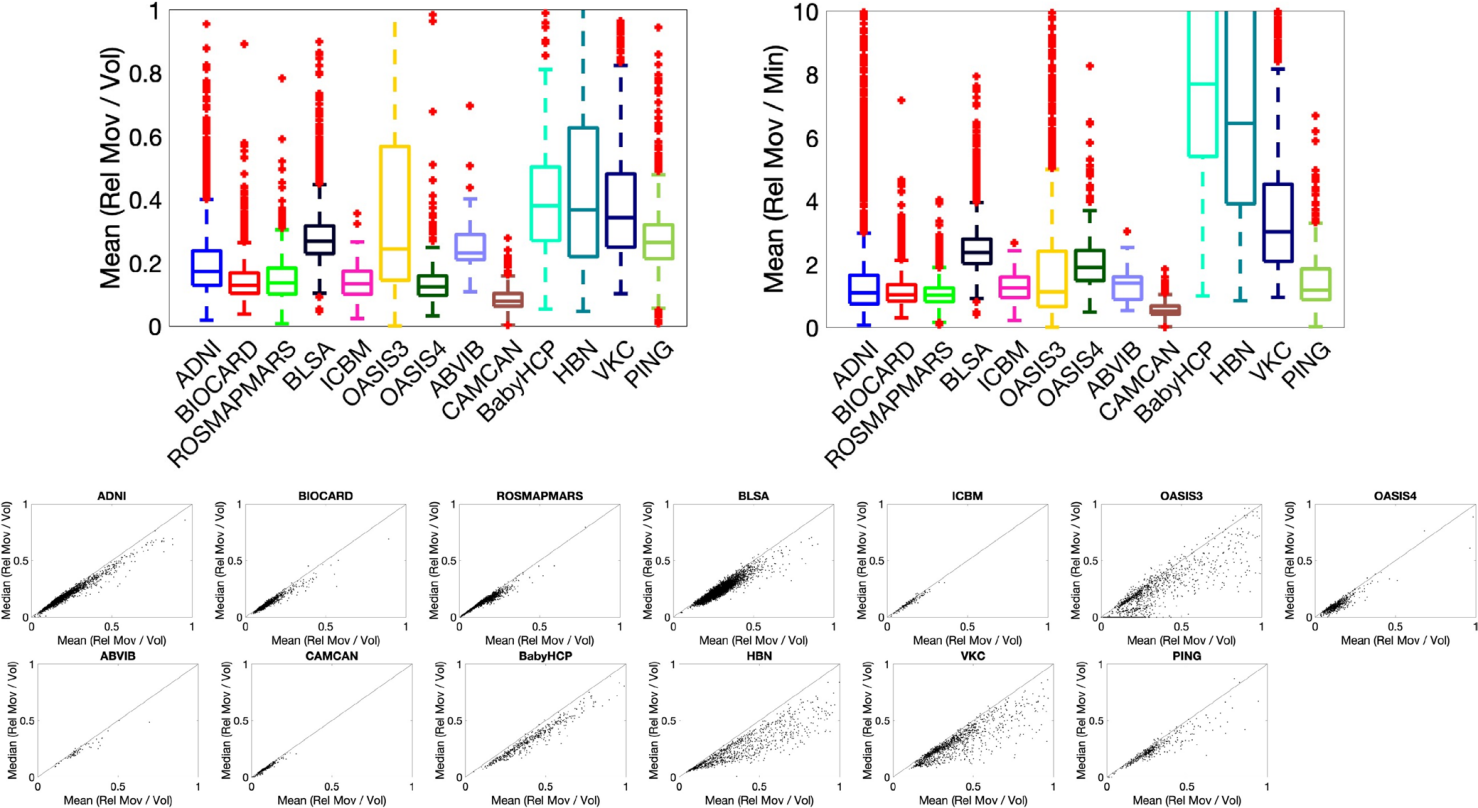
The voxel‐wise mean relative movement per minute is typically 1–2 mm, across most adult cohorts. Mean relative movement per volume (top left), mean relative movement per minute (top right), and plots of mean movement against median movement (bottom) are given for all cohorts. There are differences across cohorts (which have different acquisition parameters), baby and young childhood datasets exhibit greater motion than adult datasets. Finally, mean movement is typically greater than median movement, which suggests frequent smaller movements are more common than occasional large movements.

Figure 2 shows the mean relative movement per volume and the mean relative movement per minute for all datasets. On average, voxels in the brain moved ~0.1–0.2 mm/volume or ~ 1–2 mm/min, with differences observable across datasets. Notably higher movement was observed in the BabyHCP infant cohort, followed by the HBN and VKC datasets, which are predominantly children. The bottom half of the figure plots the mean against the median relative movement for all subjects within each dataset. In general, the mean movement was greater than the median movement, which suggests frequent smaller movements were more common than occasional large movements. Figure S3 plots the movement against number of volumes acquired and the repetition time (TR) of the scan and indicates no clear trend between motion and these acquisition parameters.

**FIGURE 3:**
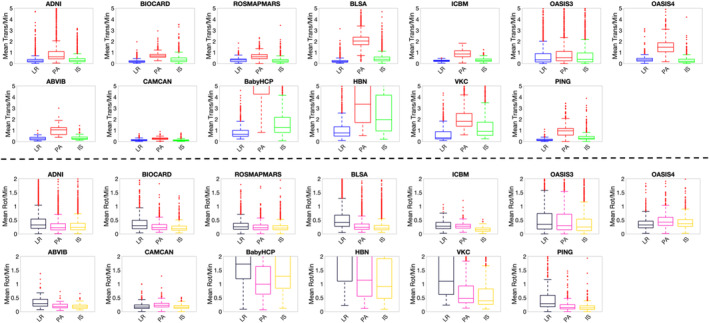
Translation is often greatest in the AP direction, with rotation often greatest around the LR axis. Plots of mean relative translation per minute (along LR, PA, IS; top), and mean relative rotation per minute (around LR, PA, and IS axes; bottom).

Figure 3 shows measures of brain translation and rotation for each dataset. For all datasets, translation was greatest in the Anterior–Posterior (AP) direction, relative to the Left–Right (LR), or Inferior–Superior (IS) directions. Measures of rotation for most datasets showed greatest rotation around the LR axis, compared to rotations around the PA and IS axes.

**FIGURE 4:**
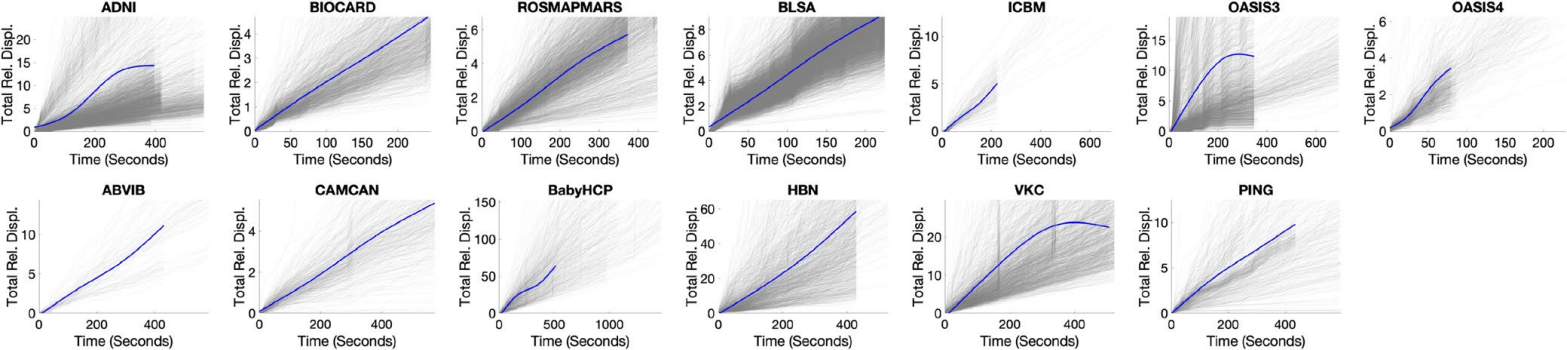
Cumulative relative displacement as the acquisition proceeds over time. For each dataset, all subjects displacement is plotted as a gray line, with the blue line showing the LOESS filtered average time course. Displacement is visually linear across most acquisitions.

The cumulative motion over time for all subjects in each dataset is plotted in Figure 4. Here, individual subjects are plotted as a transparent gray line, while the subject‐averaged motion is shown in blue. In most cases, motion largely linearly increased over the full scan duration, with few exceptions near the end of the scan, largely caused by differences in scan length within cohorts.

**FIGURE 5:**
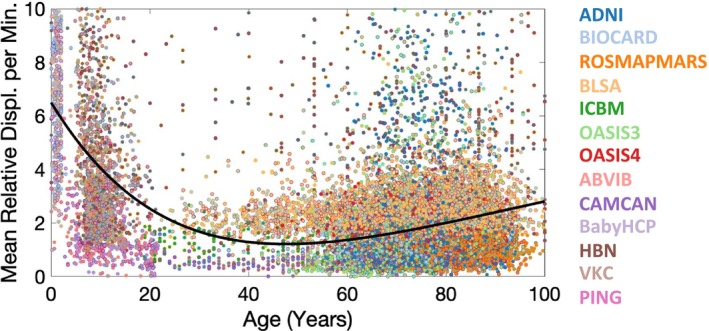
Subject motion tends to decrease into young adulthood and increases with aging. Outliers increase with age. Mean relative displacement per minute is plotted for all subjects, of all datasets, as a different color, with a simple quadratic polynomial fit shown as a yellow line.

A plot of mean displacement against age for all subjects is shown in Figure 5, with data fit to a Poisson curve, typical of many lifespan studies (Lebel et al. 2012). Motion quickly decreased cross‐sectionally during childhood into young adulthood, where it leveled off throughout adulthood. Motion then tended to increase with age, and the number of outliers with large motion also increased with age.

**FIGURE 6:**
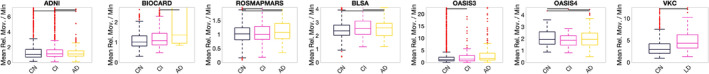
Subjects with Alzheimer's Disease (AD) tend to exhibit more motion than cognitively normal (CN) or cognitively impaired (CI). Similarly, children with learning disabilities (LD) exhibit more motion than those that are cognitively normal (CN).

Figure 6 shows motion for cognitively normal, cognitively impaired (mild cognitive impairment), and Alzheimer's subjects for each dataset. Motion tended to increase with increasing cognitive impairment, with statistically significant increases across the disability spectrum observed in most datasets. Finally, in adolescents, motion was more prevalent in volunteers with learning disabilities compared to cognitively normal volunteers.

### 3.2 Motion‐Induced Biases in Microstructure and Connectivity

**FIGURE 7:**
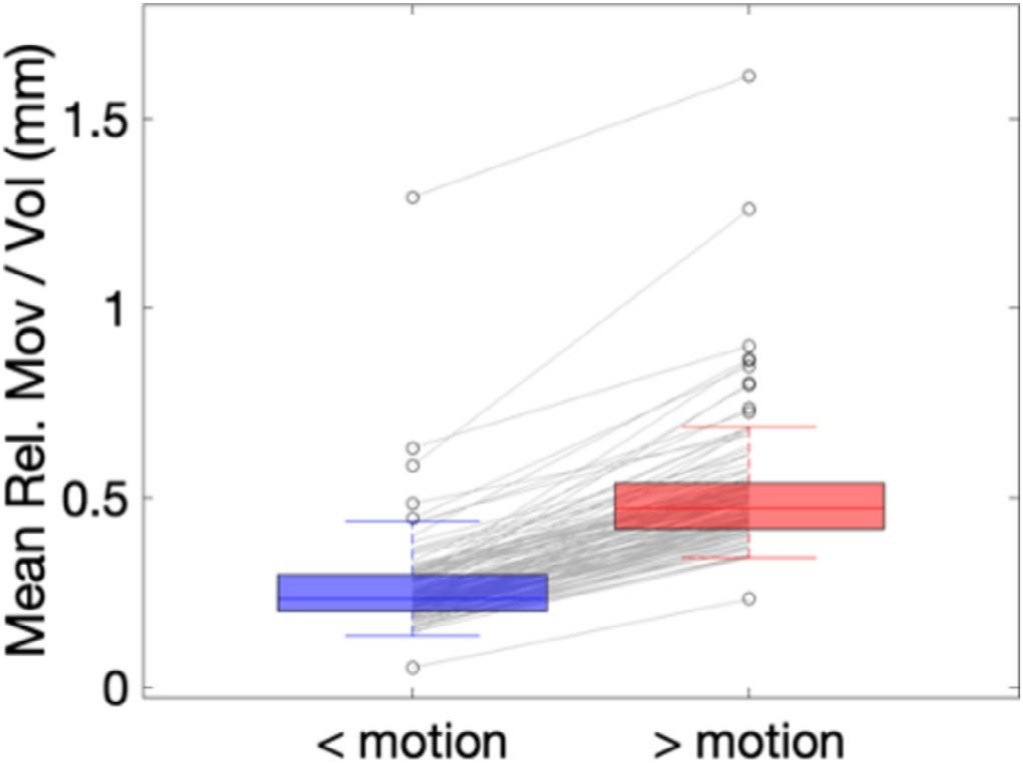
Scans and rescans of the BLSA dataset were separated into low motion and high motion scans, and the 120 individuals with the greatest difference in motion were selected for intra‐subject analysis. Mean relative motion per volume is given for both cohorts, “< motion” and “> motion”.

Figure 7 shows the mean relative movement per volume for the BLSA intra‐subject analysis where scans and rescans were assigned as low‐motion and high‐motion. By definition, high‐motion had statistically significant higher motion than low‐motion scans, by approximately ~0.3 mm/volume on average. Similar results are shown for the BIOCARD dataset Figure S4.

**FIGURE 8:**
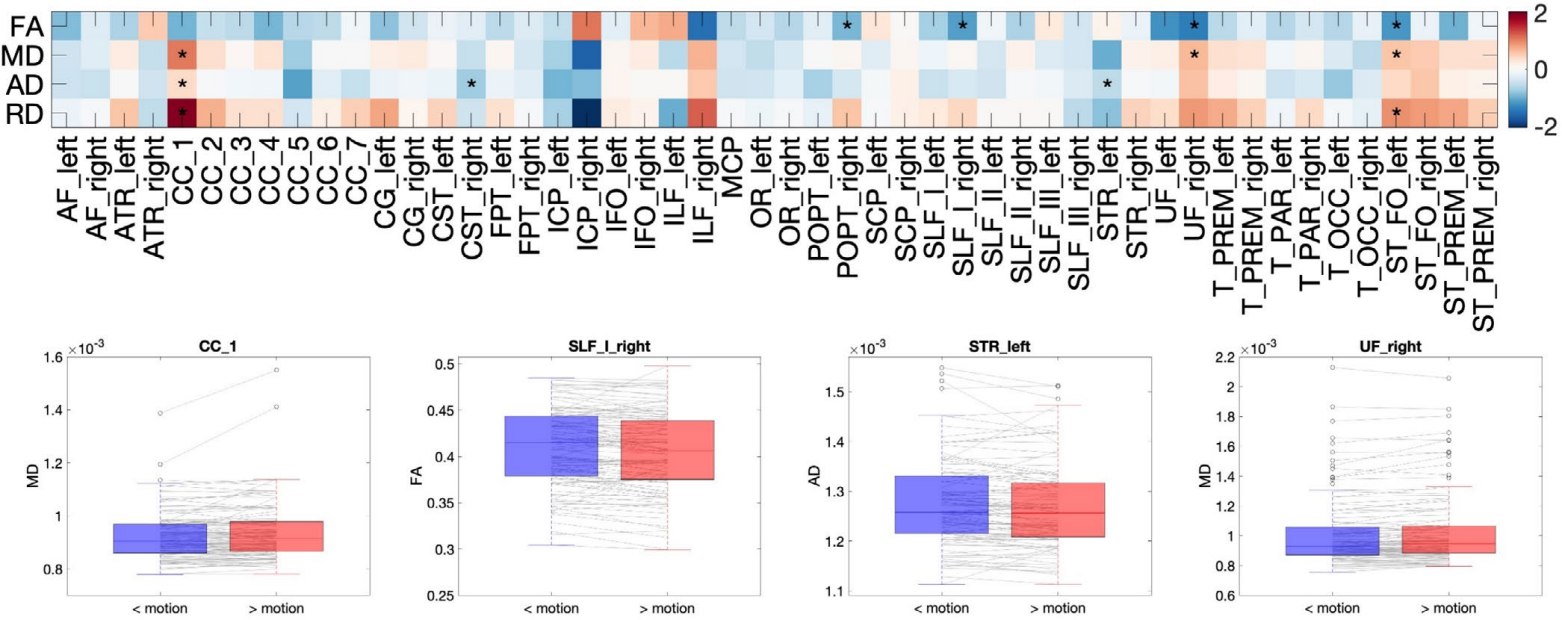
There are very few artifactual *microstructural* differences introduced by motion in the BLSA dataset. Of 200 total tests (4 *microstructural* measures × 50 pathways), 12 regions suggest *microstructural* differences between low and high motion datasets of the same subject (after FDR correction), with differences typically on the order of < 1% change. Statistically significant differences are marked with a “*”, colorbar indicates a “% change”.

Microstructural differences across all bundles are shown in Figure 8 as a measure of percent change caused by motion. First, most percent change was below 1% across all features and pathways. While trends for small decreases in FA and small increases in RD were observed, few changes were statistically significant. Of those with statistically significant bias introduced by motion, the average percent change was 0.95%, an effect well below typical detection thresholds associated with diffusion analysis. Similar results were found for the BIOCARD dataset, in which there were 0 statistically significant differences between cohorts (Figure S5).

**FIGURE 9:**
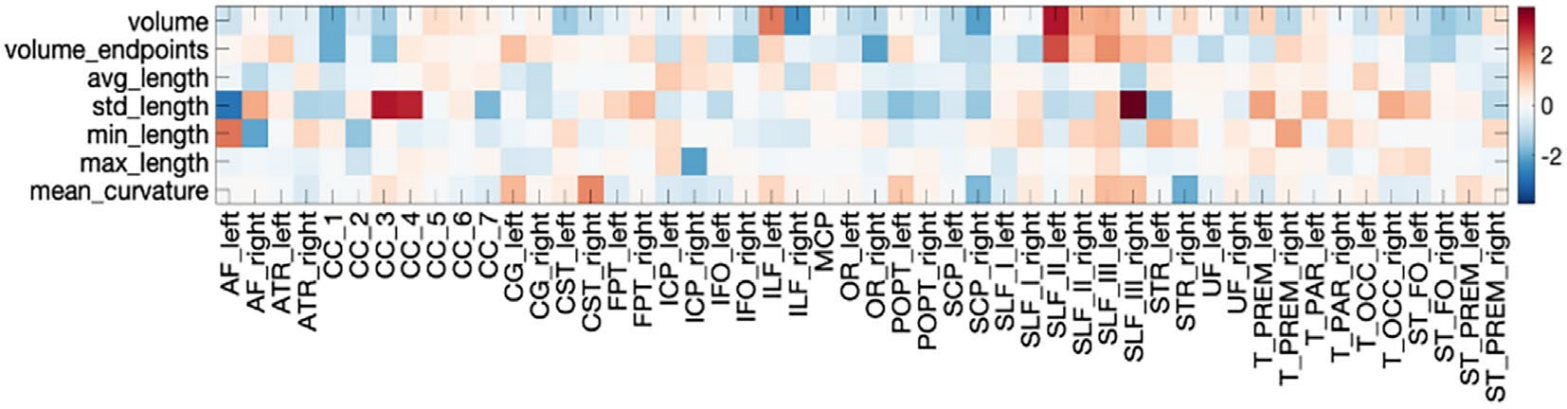
There are no artifactual macrostructural differences introduced by motion in the BLSA dataset. Of 350 total tests (7 *macrostructural* measures × 50 pathways), no regions suggest macrostructural differences between low and high motion datasets of the same subject (after FDR correction). Statistically significant differences are marked with a “*” (note there are no statistically significant differences), colorbar indicates a “% change”.

Macrostructural differences across all bundles are shown in Figure 9. There were no statistically significant differences between the low‐ and high‐motion cohorts, and thus no artifactual differences introduced by motion. No trends were apparent for any features. Similar results were found for the BIOCARD dataset (Figure S6).

**FIGURE 10:**
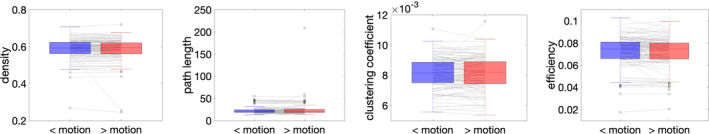
There are no artifactual *connectomic* differences introduced by motion in the BLSA dataset. No *connectomic* measures suggest differences between low and high motion datasets of the same subjects.

Connectomic differences between low‐ and high‐motion datasets are shown in Figure 10. For all 5 investigated network measures, no statistically significant differences were found, thus no artifactual differences were introduced by motion. Similar results were found for connectomics in the BIOCARD dataset (Figure S7).

A final along‐fiber quantification, which tests for statistically significant differences along entire pathways, confirmed the results of Figure 7, showing few, and sparse, differences caused by motion (Figures S8 and S9).

### 3.3 Structural Connectivity Differences Between Movers and Non‐Movers

**FIGURE 11:**
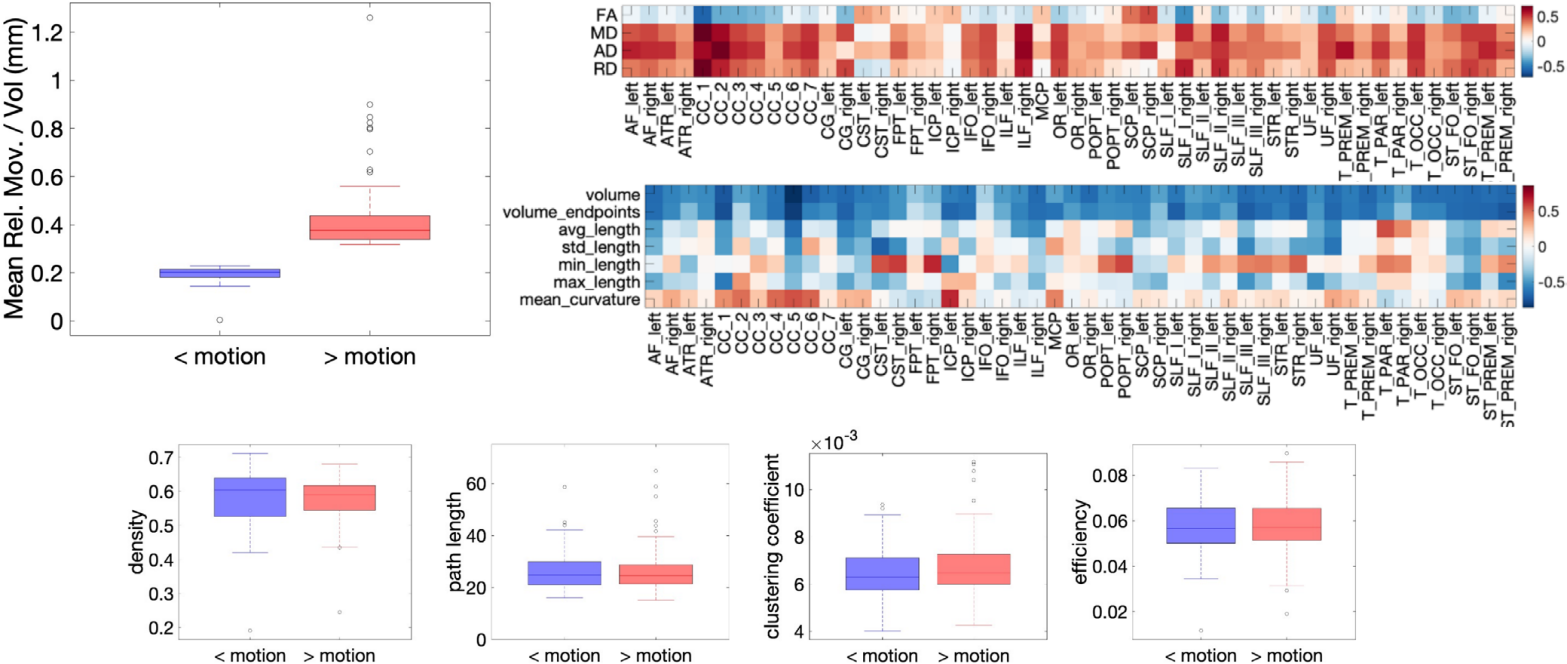
There are no microstructural, macrostructural, or connectomic differences between movers and non‐movers in the evaluated data sets. (Top left) By definition, movers (> motion) has greater movement than non‐movers (< motions), (top right) effect size for microstructural measures, (middle right) effect size for macrostructural measures, (bottom) box plots for connectomic measures. Statistically significant differences are marked with a “*” (note there are no statistically significant differences).

Figure 11 summarizes the results assessing the differences between non‐movers and movers. First, by definition, movers had greater motion per volume than non‐movers. Second, while there were trends in microstructure measures (decreased FA and increased diffusivities for movers), no statistically significant microstructural differences between movers and non‐movers were found. Similarly, while measures of volume and length tended to decrease for movers, there were no statistically significant macrostructural differences. Finally, there were no differences in connectome descriptors between movers and non‐movers.

## 4 Discussion

Motion in the MR scanner can have substantial impacts on quantitative measures of brain structure and function and confound interpretation of results and scientific conclusions. Here, we study motion in studies using diffusion MRI. We have three primary conclusions. First, volunteers in the scanner move ~1–2 mm per minute, with motion most common as a rotation around the LR axis. Second, motion in the range studied in our work is well‐corrected for in modern preprocessing pipelines and does not introduce significant biases in quantitative analysis. Third, no microstructural, macrostructural, or connectomic‐based measures of differences (as measured by diffusion MRI) were found between volunteers who move and those who do not.

### 4.1 Characterizing Motion During Diffusion MRI Scans

While movement in diffusion MRI is a well‐known challenge (Anderson and Gore 1994), a thorough retrospective characterization of this motion, particularly across several cohorts, has not been performed. We characterize motion across several large cohorts and consortiums, across a large age range and find that on average, volunteers in the scanner move ~1–2 mm per minute. This number represents the brain‐averaged voxel‐wise displacement and is just less than the size of a typical diffusion MRI voxel per minute.

We find frequent smaller movements more common than occasional large movements, which is useful knowledge for designing preprocessing pipelines and parameter settings, as motion typically occurs in a “drift” style away from the first collected volume, with sparse jumps from volume to volume. There are also meaningful site differences—meaning that in addition to scanner effects, choices in subject positioning and immobilization strategies, as well as scan protocol, affect the motion observed in medical images. Our preliminary analysis found no clear trends related to scan settings (thus, we do not give recommendations on an optimal scan length for minimizing motion), but further analysis should investigate how choices in scan length, number of volumes, and time between scans (TR) influence observed motion and subsequent artifacts. Subject preparation for each cohort is less detailed in the literature. There are several preparation strategies that aim to increase patient comfort—including maintaining airflow throughout the bore and/or blankets to maintain comfortable body temperature, minimizing peripheral nerve stimulation (an acquisition consideration), cushion/towel to support the neck, frequent communication throughout the scan, comfortable combination of headphones + padding + coil—it would require dedicated studies to investigate each strategy independently, although motion‐mitigation efficacy will likely depend on interactions between these and acquisition strategies.

Translation occurs most in the AP direction, while rotation occurs round the LR axis. These are possibly the least constrained directions of motion, as earpads/constraints prevent LR translation and SI rotation. While intuitive, this observation has not been well described in literature, and we show that the direction of motion generalizes across cohorts and scan sites. We note that because of the concurrent estimation of motion and eddy currents, there is an ambiguity between any eddy current field and a translation in the phase encode direction, which is most often the AP direction, meaning this is indeed the least well‐quantified direction of motion.

Knowledge of the relative motion of scan time may be useful in designing acquisitions and determining appropriate scan lengths. We hypothesized that motion would increase as the scan proceeds. However, qualitatively, this does not seem to be the case, as motion appears largely linear throughout the entire protocol.

Finally, not surprisingly, we find that infants and children exhibit the greatest motion during the scan, typically 3‐8x as much as a typical young adult cohort. Leveling off during young adulthood, motion again increases with age. Moreover, outliers become more frequent above the age of 60. These results emphasize the particular need for QA in these cohorts, and motivated the 2nd and 3rd parts of the current manuscript in which we explore whether these motion effects must be accounted for in statistical modeling to ensure spurious, non‐biological differences are not created. Similar results are observed in those with cognitive impairment and developmental delays, with motion increasing along the impairment spectrum.

We expect these results to generalize to across data/contrasts, as there is little fundamental difference to the subject between diffusion MRI and functional MRI, for example, especially as both are typically acquired with an EPI readout. Future studies should confirm similar results in functional imaging, and investigate the effects of motion corruption on microstructure and connectivity estimates derived from diffusion MRI.

### 4.2 Modern Preprocessing Pipelines Mitigate Biases in Microstructure and Macrostructure

Using data from children with autism and typically developing children, Yendiki et al. (Yendiki et al. 2014) show that group differences in motion can introduce spurious group differences in microstructure measures. They extended this analysis to comparisons with control groups in which no diffusivity differences were expected, finding group differences of up to 6% in various measures, with statistically significant effects in often as many as 5+ studied white matter pathways. For this reason, they suggest including motion as a nuisance regressor in any diffusion‐based analysis. We aimed to extend this study, but using state‐of‐the‐art preprocessing pipelines, with advances in artifact and outlier detections to investigate whether these spurious group differences are still observed.

Our main result is that modern preprocessing pipelines effectively mitigate these biases. While we do see trends for decreased FA and increased RD due to motion, these results are not statistically significant, and frequently under a 1% change. This is an optimistic outcome, well‐validating these preprocessing pipeline's ability to not only correct motion, but ensure scans have the same geometry (susceptibility distortion correction) and signal intensities (outlier detection and replacement). Beyond this, tractography‐based macrostructure measures of tract volumes, lengths, and areas are well preserved with motion, and biases are not introduced in connectomic graph theory measures. These results generalized to two datasets with different scanners and acquisitions. While there are not apparent biases, as in (Yendiki et al. 2014), we still advocate for the inclusion of motion as a nuisance regressor in group studies, either as averaged voxel‐wise displacement or 6° of translations and rotations common in functional studies. While we cannot give concrete guidance on a threshold for inclusion/exclusion criteria (as we did not exclude any scans in the intra‐subject analysis, and still find no biases), we do strongly further advocate visually inspecting the data, including raw data, derived indices, and parameter fits, as commonly implemented in quality control packages (Cai et al. 2021; Oguz et al. 2014; Esteban et al. 2017). As discussed in Yendiki et al. (Yendiki et al. 2014), more poor quality datasets (in children with autism and in typically developing children) were excluded by visual analysis than by any threshold on motion parameters. Possible future investigations may utilize bootstrapping in empirically collected data to investigate nuisance regressors and their relationships to biases and variance in derived measures (Sairanen and Andersson 2023). This is still not common practice; however, many quality assurance and quality control packages (Cai et al. 2021; Bastiani et al. 2019; Haddad et al. 2019) often include and display these values, providing an easy measure to implement to mitigate false positive findings.

Our results agree quite nicely with a recent study by Cieslak et al. (Cieslak et al. 2024). Using a realistic simulated dataset, they find that diffusion preprocessing pipelines are highly accurate at correcting motion throughout the scan, but are impacted by denoising and image acquisition settings. While our study does not have a ground truth for which to compare the motion estimates themselves, we find that after preprocessing, the diffusion‐derived indices do not suffer from biases traditionally associated with motion (such as increased RD and decreased FA) (Anderson and Gore 1994; Chang, Jones, and Pierpaoli 2005). Thus, our study extends the findings of Ciesklak et al. (Cieslak et al. 2024) to empirically acquired data, confirming that preprocessing methods do well at reducing artifacts caused by motion.

### 4.3 Structural Connectivity Differences Between Movers and Non‐Movers

Subject motion has been associated with executive function (lower cognition and poorer inhibition control) (Hausman et al. 2022), cognitive status (Geerligs et al. 2017), weight, blood pressure, memory, and sleep issues (Bolton et al. 2020), and increases with age (Geerligs et al. 2017; Madan 2018; Pardoe, Kucharsky Hiess, and Kuzniecky 2016). Further, head motion has been shown to be significantly heritable, which suggests it may be a neurobiological trait (Couvy‐Duchesne et al. 2014). Indeed, some neurobiological features of head motion have been uncovered using functional MRI, for example increased distance connectivity between cognitive associated regions mainly in the default mode network (Zeng et al. 2014). Here, we hypothesized that there may be structural biological differences associated with motion in the form of changes in tissue microstructure or the shape and geometry of different fiber pathways. While there were general trends where movers exhibited decreased anisotropies, increased diffusivities, and decreased volumes (the same trends typically observed in pathologies or aging), these were not statistically significant across any pathways nor any features. Thus, while head motion in the scanner may be a neurobiological trait, it seems driven by function rather than structure (at the scale measured by diffusion MRI).

### 4.4 Limitations and Future Directions

There are several limitations and avenues for future exploration. First, we focus our analysis on degrees of motion that can be characterized using retrospective motion correction algorithms. Subjects with very drastic head movements, or very high numbers of outlier slices, may not provide accurate measures of motion. The particular algorithm we chose (FSL's EDDY) can work quite well up to a certain amount of outliers (~10% in the case of high angular resolution acquisitions (Sairanen and Andersson 2023; Andersson et al. 2016; Cieslak et al. 2024)), and the maximum tolerable frequency of outliers as a function of gradient directions is unknown. For example, our intra‐subject analysis may not generalize to our datasets with significantly more motion (HBN, babyHCP). To map all degrees and magnitudes of head motion it is probably necessary to use additional sensors or motion tracking sequences (van Niekerk et al. 2022). Next, clearly different cohorts and consortiums have different levels of motion, which cannot be uniquely attributed to either volunteer/scanner preparation or image quality and is likely a combination of both factors.

Additionally, different gradient schemes may be more affected by motion than others. Similarly, we explore DTI, which may be quite robust to outliers and SNR, particularly with 20–30+ directions, and more advanced signal representations (e.g., Diffusion Kurtosis Imaging (Coutu et al. 2014) or multi‐compartment models (Novikov, Kiselev, and Jespersen 2018)) may be differentially biased by head motion. Interestingly, a recent study by Pines et al. (Pines et al. 2020) show that models leveraging multi‐shell information may be less sensitive to confounding effects of motion than single shell data. In contrast to our results, they find that white matter‐averaged FA, MD, and RD had small, but significant, correlations with motion‐metrics (*p* = 0.017, 0.013, and 0.006, respectively). The contrasting results could be due to higher motion and range of motion in their cohort (children versus our BLSA/BIOCARD cohorts), increased sensitivity/precision of metrics (averaged WM microstructure versus our bundle‐based measures), or statistical analysis (cross‐sectional controlling for age and sex versus our same‐subject analysis but with low and high motion scans, but where we know the biology does not change within the scan session). Regardless, their results are clear that there are different sensitivities to motion due to different acquisition schemes (directions, b‐values) and modeling strategies.

Next, we investigated only global connectomic measures describing features of the connectome as a single scalar value. While we do not intend to do a deep dive into edge‐ and node‐based measures due to the combinatorial combinations of edge‐strength weighting schemes and normalizations, an analysis of our intra‐subject dataset (same subjects, no biological differences) found 0 edges (weighted by SIFT2‐derived cross‐sectional area weights) that were statistically significantly different due to motion (Figure S10). Our inter‐subject analysis (low and high motion cohorts) found 6% of edges show a statistically significant difference between cohorts (Figure S11). This is in line with previous works (Baum et al. 2018) studying the impact of head motion on structural connectivity, where 12% of edges were impacted by motion (Figure 2 of (Baum et al. 2018)) (and more when edges were weighted by FA), although there was a statistically significant correlation between motion and edge weights for all edges. Again, differences in choices of streamline propagation and filtering (our choice of anatomical constraints and SIFT2 filtering) could explain discrepancies. Thus, a thorough exploration of effects on edge and node‐based measures, and choices in acquisition, preprocessing, and streamline filtering, is warranted in future studies. Similarly, the effects of motion on image denoising algorithms (and their influence on preprocessing pipelines) should be studied to fully optimize diffusion preprocessing.

## 5 Conclusion

Head movement in the scanner induces artifacts and biases in MR images. We characterized motion during diffusion MRI scans in several large multi‐center cohorts and found (1) subjects typically move 1–2 mm/min with most motion as translation in the AP direction and around the RL axis (2) modern preprocessing pipelines can effectively mitigate motion to the point where biases are not detectable with current analysis techniques, and (3) there are no apparent differences in microstructure or macrostructural connections in volunteers who exhibit high motion versus those that exhibit low motion. Characterizing motion magnitude and directions, as well as motion correlates, informs and improves motion mitigation strategies and image processing pipelines.

## Supporting information


**Data S1:** Supporting Information.

## Data Availability

Data sharing is not applicable to this article as no new data were created or analyzed in this study.
