# Supplementary material for: Head Motion in Diffusion Magnetic Resonance Imaging: Quantification, Mitigation, and Structural Associations in Large, Cross‐Sectional Datasets Across the Lifespan
Source: Hum Brain Mapp. 2025 Feb 11;46(3):e70143. doi: 10.1002/hbm.70143 (PMC11814480; doi:10.1002/hbm.70143)
Supplement: Supplementary file 1 — Data S1: Supporting Information. [file HBM-46-e70143-s001.docx]

**Supplementary Material for:**

**Head motion in diffusion MRI: Quantification, mitigation, and structural associations in a large, cross-sectional dataset of lifespan and special populations**

Kurt G Schilling^1,2^, Karthik Ramadass^6^, Viljami Sairanen^3,4^, Michael E Kim^6^, Francois Rheault^9^, Nancy Newlin^6^, Tin Nguyen^7,8,2^, Laura Barquero^7,8^, Micah D’archangel^7,8^, Chenyu Gao^5^, Ema Topolnjak^6^, Nazirah Mohd Khairi^6^, Derek Archer^12,13^, Lori L. Beason-Held^14^, Susan M. Resnick^14^, Timothy Hohman^11,12,13^, Laurie Cutting^7,8^ , Julie Schneider^17^, Lisa L. Barnes^17^, David A. Bennett^17^, Konstantinos Arfanakis^18,19,20^, Sophia Vinci-Booher^10^, Marilyn Albert^21^, The BIOCARD Study Team^16^, The Alzheimer’s Disease Neuroimaging Initiative (ADNI)^15^, and Aging Brain: Vasculature, Ischemia, and Behavior (ABVIB), Daniel Moyer^5^, Bennett A Landman^1,2,5,6^

1. Department of Radiology & Radiological Sciences, Vanderbilt University Medical Center, Nashville, TN, USA
2. Vanderbilt University Institute of Imaging Science, Nashville, TN, USA
3. Baby Brain Activity Center, Children’s Hospital, Helsinki University Hospital and University of Helsinki, Helsinki, Finland
4. Department of Radiology, Kanta-Häme Central Hospital, Hämeenlinna, Finland
5. Department of Electrical and Computer Engineering, Vanderbilt University, Nashville, TN, USA
6. Department of Computer Science, Vanderbilt University, Nashville, TN, USA
7. Special Education, Psychology & Human Development, Radiology, Pediatrics, and Computer and Electrical Engineering
8. Vanderbilt Kennedy Center, Nashville, TN, USA
9. Medical Imaging and Neuroinformatic (MINi) lab, Universite de Sherbrooke, Sherbrooke, Quebec, Canada
10. Department of Psychology and Human Development, Peabody College, Vanderbilt University, Nashville, TN, USA
11. Department of Neurology, Vanderbilt University, Nashville, TN, USA
12. Vanderbilt Memory & Alzheimer's Center, Vanderbilt University Medical Center, Nashville, Tennessee, USA
13. Vanderbilt Genetics Institute, Vanderbilt University Medical Center, Nashville, Tennessee, USA
14. Laboratory of Behavioral Neuroscience, National Institute on Aging, National Institutes of Health, Baltimore, MD, USA
15. Data used in preparation of this article were obtained from the Alzheimer’s Disease Neuroimaging Initiative (ADNI) database (adni.loni.usc.edu). As such, the investigators within the ADNI contributed to the design and implementation of ADNI and/or provided data but did not participate in analysis or writing of this report. A complete listing of ADNI investigators can be found at: <http://adni.loni.usc.edu/wp-content/uploads/how_to_apply/ADNI_Acknowledgement_List.pdf>
16. Data used in preparation of this article were derived from BIOCARD study, supported by grant U19 – AG033655 from the National Institute on Aging. The BIOCARD study team did not participate in the analysis or writing of this report, however, they contributed to the design and implementation of the study. A listing of BIOCARD investigators can be found on the BIOCARD website (on the ‘BIOCARD Data Access Procedures’ page, ‘Acknowledgement Agreement’ document).
17. Rush Alzheimer's Disease Center, Rush University Medical Center, Chicago, IL
18. Department of Biomedical Engineering, Illinois Institute of Technology, Chicago, IL
19. Rush Alzheimer’s Disease Center, Rush University Medical Center, Chicago, IL
20. Department of Diagnostic Radiology, Rush University Medical Center, Chicago, IL
21. Department of Neurology, Johns Hopkins University School of Medicine, Baltimore, MD

**Supplementary Figure 1.** Histogram of datasets, sample size, and age ranges. We used 13 large consortium datasets to study subject motion across a population aged 0-100 years. Specifically, we chose diffusion MRI data which involved collection of multiple volumes over several minutes and allowed characterizing motion: ADNI (N=2521) [16], BIOCARD (N=974) [17], ROSMAP (N=1973) [18], BLSA (N=5424) [19], ICBM (N=140) [20], OASIS3 (N=1794) [21], OASIS4 (N=603) [22], ABVIB (N=63) , CAMCAN (N=305) [23], BabyHCP (N=494) [24], HBN (N=1006) [25], PING [26] (N=431), and data collected by the Vanderbilt Kennedy Center (VKC) (N=149).

**Supplementary Figure 2.** Datasets ADNI, BIOCARD, ROSMAPMARS, BLSA, OASIS3, and OASIS4 included cognitively normal (CN), cognitively impaired (CI), and subjects with Alzheimer’s Disease (AD). Finally, the VKC dataset included children who all scored within the typically developing range on IQ tests; however, some were at risk for or had learning disabilities (LD) and/or other developmental disorders (e.g., ADHD). Figure shows boxplots of sample size and cohort size for these datasets.


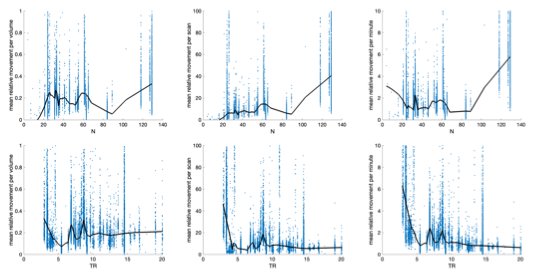


**Supplementary Figure 3.** There are no clear trends between motion and acquisition parameters number of volumes (N) and TR. (Top) Movement per volume, movement per scan, movement per minute plotted against the number of diffusion volumes (N). (Bottom) Movement per volume, movement per scan, movement per minute plotted against TR.


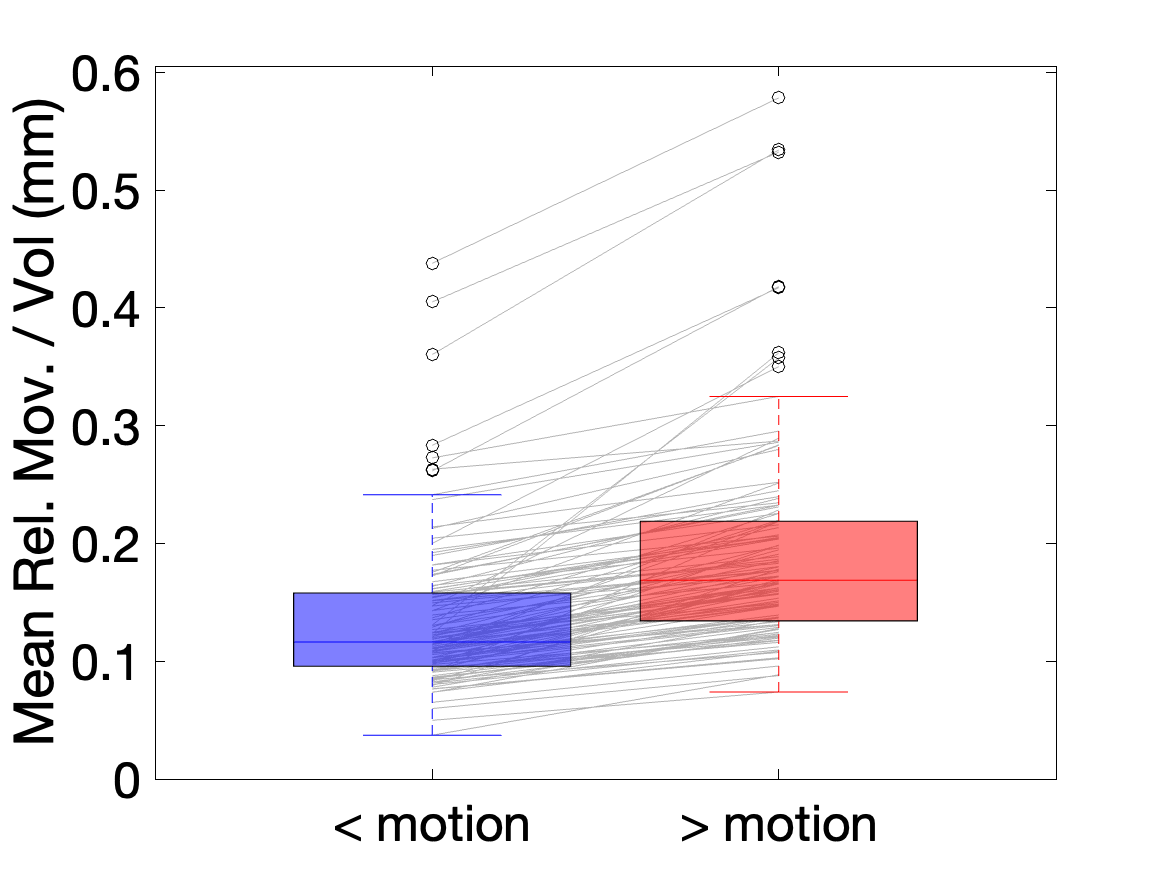


**Supplementary Figure 4.** Scans and rescans of the BIOCARD dataset were separated into low motion and high motion scans, and the 120 individuals with the greatest difference in motion were selected for intra-subject analysis. Mean relative motion per volume is given for both cohorts, “< motion” and “> motion”. This figure parallels Figure 7 in the main manuscript (BLSA dataset).


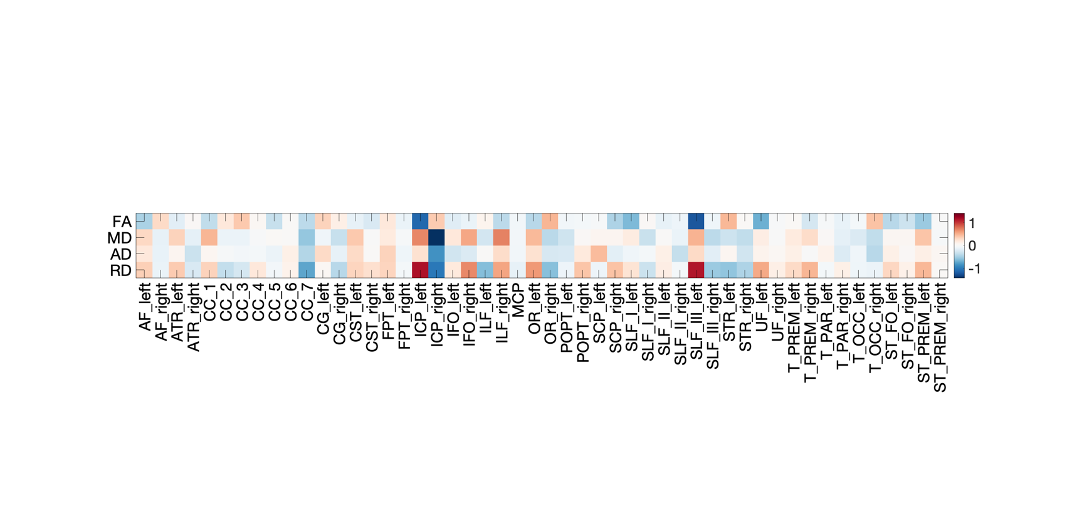


**Supplementary Figure 5.** There are no artifactual *microstructural* differences introduced by motion in the BIOCARD dataset. Of 200 total tests (4 *microstructural* measures x 50 pathways), no regions suggest *microstructural* differences between low and high motion datasets of the same subject (after FDR correction), with differences typically on the order of <1% change. This figure parallels Figure 8 in the main manuscript (BLSA dataset). Statistically significant differences are marked with a “*” (note there are no statistically significant differences).


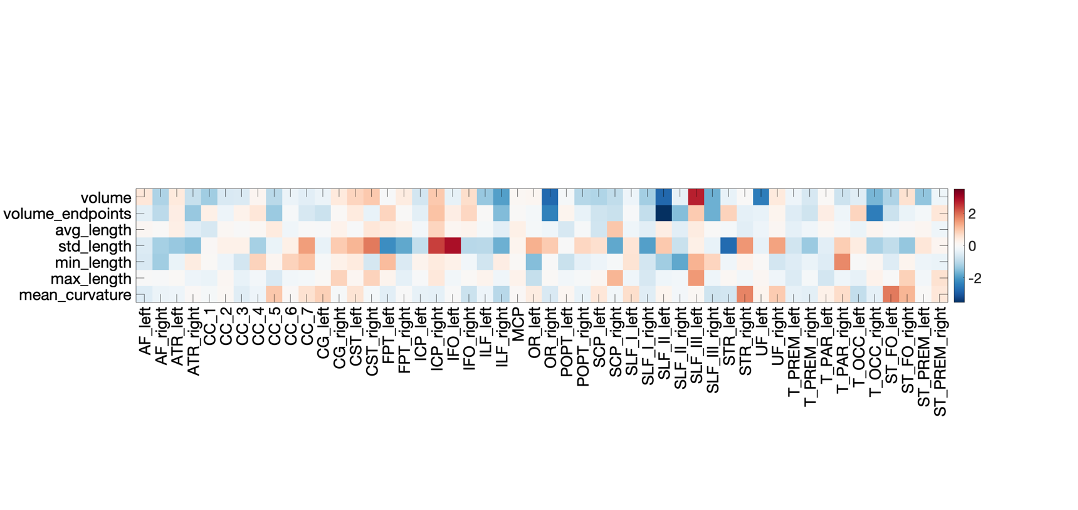


**Supplementary Figure 6.** There are no artifactual macrostructural differences introduced by motion in the BIOCARD dataset. Of 350 total tests (7 *macrostructural* measures x 50 pathways), no regions suggest macrostructural differences between low and high motion datasets of the same subject (after FDR correction). This figure parallels Figure 9 in the main manuscript (BLSA dataset). Statistically significant differences are marked with a “*” (note there are no statistically significant differences).


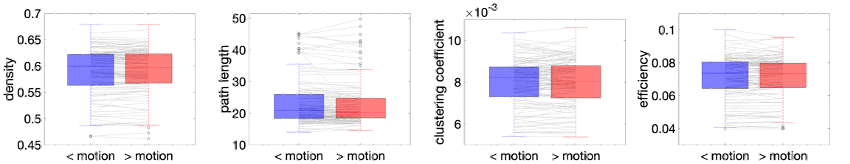


**Supplementary Figure 7.** There are no artifactual *connectomic* differences introduced by motion in the BIOCARD dataset. No *connectomic* measures suggest differences between low and high motion datasets of the same subjects. This figure parallels Figure 10 in the main manuscript (BLSA dataset).





**Supplementary Figure 8.** There are few artifactual *along fiber quantification* differences introduced by motion in the BLSA dataset. For each pathway, plots show microstructural differences between low and high motion datasets of the same subjects sampled on 100 points along the pathway, as well as % change (or % difference) between the two cohorts.





**Supplementary Figure 9.** There are few artifactual *along fiber quantification* differences introduced by motion in the BIOCARD dataset. For each pathway, plots show microstructural differences between low and high motion datasets of the same subjects sampled on 100 points along the pathway, as well as % change (or % difference) between the two cohorts.


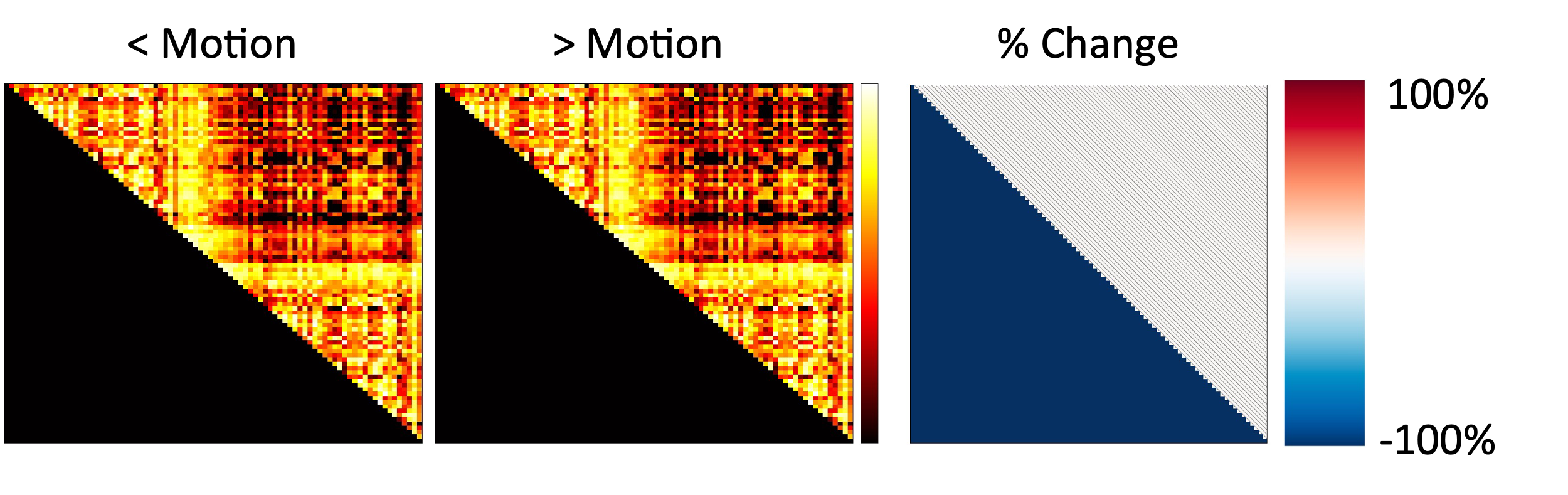


**Supplementary Figure 10.** There are no artifactual connectomic differences in edge-based connectivity strength introduced by motion in the BLSA dataset. The subject-averaged connectome is shown for both the low-motion (left) and high-motion (right) cohorts (the cohorts are derived from the same subjects). Statistically significant changes due to motion are colored as a Percent Change (right) after Wilcoxin Signed Rank tests and FDR correction. Note that there are no statistically significant changes in edge-based connectivity so all % change is shown as white. Connectomes are shown as the apparent fiber density of connections derived using MRTrix3 SIFT2 filtering and shown on a log-scale to highlight large contrast range. Similar results are obtained with and without filtering (i.e., number of streamlines for each edge).

**Supplementary Figure 11.** There are few artifactual connectomic differences in edge-based connectivity strength between movers and non-movers in the evaluated datasets. Cohorts are derived from highest and lowest movement estimates from the BLSA datasets, and by definition, movers have greater movement than non-movers (see Figure 11 in the main text). The subject-averaged connectome is shown for both the non-movers (left) and movers(right). Statistically significant changes due to motion are colored as a Percent Change (right) after Wilcoxin Rank Sum tests and FDR correction. Connectomes are shown as the apparent fiber density of connections derived using MRTrix3 SIFT2 filtering and shown on a log-scale to highlight large contrast range. Similar results are obtained with and without filtering (i.e., number of streamlines for each edge).
